# First examples of organosilica-based ionogels: synthesis and electrochemical behavior

**DOI:** 10.3762/bjnano.8.77

**Published:** 2017-03-29

**Authors:** Andreas Taubert, Ruben Löbbicke, Barbara Kirchner, Fabrice Leroux

**Affiliations:** 1 Institute of Chemistry, University of Potsdam, D-14476 Potsdam, Germany; 2 Mulliken Center for Theoretical Chemistry, Institut für Physikalische und Theoretische Chemie, Universität Bonn, Beringstraße 4+6, D - 53115 Bonn, Germany; 3 Inorganic Materials, Institut de Chimie de Clermont-Ferrand (ICCF) - UMR 6296, Université Blaise Pascal, Chimie 5, Campus des Cézeaux, 24 avenue des Landais, BP 80026 63171 Aubière Cedex, France

**Keywords:** ionic liquids, ionogels, organosilica, proton conductivity

## Abstract

The article describes the synthesis and properties of new ionogels for ion transport. A new preparation process using an organic linker, bis(3-(trimethoxysilyl)propyl)amine (BTMSPA), yields stable organosilica matrix materials. The second ionogel component, the ionic liquid 1-methyl-3-(4-sulfobutyl)imidazolium 4-methylbenzenesulfonate, [BmimSO_3_H][PTS], can easily be prepared with near-quantitative yields. [BmimSO_3_H][PTS] is the proton conducting species in the ionogel. By combining the stable organosilica matrix with the sulfonated ionic liquid, mechanically stable, and highly conductive ionogels with application potential in sensors or fuel cells can be prepared.

## Introduction

Ionic liquids (ILs), that is, substances solely composed of ionic species have been studied for virtually every application from organic synthesis to lubrication and battery technology [1–4]. A particularly promising field of IL-based materials is the general area of advanced energy technology, such as proton-exchange membrane (PEM) or alkaline fuel cells, solar cells, or various battery types [1,5–7]. ILs offer, unlike conventional solvents and substances, easy access to virtually unlimited structural diversity by simple variation of the respective ions. This facilitates the tailoring of their properties, e.g., viscosity, ionic conductivity, solubility, or melting and glass points and therefore makes ILs perfect media for task-specific applications [1,4]. Additionally, compared to many systems based on molecular solvents, ILs often offer improved safety of a device by way of their low vapor pressure and low flammability [8].

As a result, ILs have been investigated as advanced electrolytes to replace traditional aqueous or organic electrolytes in batteries and fuel cells [5–7]. Among others, ILs are interesting for intermediate temperature fuel cells operating above ca. 80 °C. At this point, conventional Nafion membranes dry out and lose the ability for proton conduction [9]. Due to their relatively high thermal stability, high ionic conductivity, and low vapor pressure, many ILs can overcome this temperature limit and provide access to proton transporting membranes that can operate in the anhydrous state up to ca. 200 °C [5,10].

However, to use ILs in electrochemical devices such as fuel cells, they need to be immobilized in a viable matrix. Materials resulting from the combination of a support (silica, polymer, colloidal particles, carbon nanotubes, or gelators) and an IL are called ionogels (IGs) or ion-gels [11–13].

Several research groups have put forward approaches towards mechanically stable IGs and studied their electrochemical properties. Gayet et al. made silica/poly(methyl methacrylate)-based IGs using 1-butyl-3-methylimidazolium bis(tri-fluoromethanesulfonyl)imide, [Bmim][NTf_2_], to obtain an IG membrane with high ionic conductivity [14–15]. Néouze et al. studied IGs from the same IL and a silica matrix. The IGs exhibit rather high conductivities of up to 10^−3^ S·cm^−1^. This is close to the conductivity of the pristine IL indicating that the confinement of the IL into a matrix does not significantly affect the conductivity [11,16–17]. Again using the same IL, Martinelli and coworkers have provided a set of interesting studies on how the ion flexibility and dynamics affect the conductivity in silica nanoparticle-based IGs and related systems [18–22]. Horowitz and Panzer synthesized mechanically compliant silica-based IGs. These IGs also showed unusually high IL loadings of up to 94% [23–24].

Delahaye et al. used the sulfonated IL 1-methyl-3-(3-sulfopropyl)-imidazolium *para*-toluenesulfonate, [PmimSO_3_H][PTS], and a silica-based matrix to synthesize IGs with conductivities of 10^−2^ and 10^−3^ S·cm^−1^ in the hydrated and anhydrous state, respectively [25]. Negre et al. reported IG-based supercapacitors that can be operated over a 3 V cell voltage window. Moreover, the supercapacitor has a capacitance up to 90 F/g at room temperature [26]. Ameri et al. made IG transistors using graphene [27] for low voltage operation.

Using a slightly different approach, Hesemann and coworkers and Néouze and coworkers incorporated IL-like functionalities into periodic mesoporous organosilicas (PMOs) [28–29] and into silica nanoparticle networks [30–33], respectively. Although no IGs were made from either of these materials, they are quite similar to the organosilica matrix materials used in the current work.

The current article is the first account of organosilica-based IGs. The IGs combine the advantageous (mechanical) properties of organosilica hosts and the IL 1-methyl-3-(3-sulfobutyl)imidazolium *para*-toluenesulfonate, [BmimSO_3_H][PTS], which has a high ionic conductivity and can be synthesized in high purity in a very simple reaction at near-quantitative yields. The materials have two main advantages over existing proton-conducting IGs (PIGs). First, the silica matrix shows a better mechanical stability than neat silica due to the organic bridging moieties present in the organosilica host. Second, the organic moiety of these organosilanes contains nitrogen atoms, which can reversibly be protonated and should thus also contribute to the ionic conductivity of the PIGs. A further synthetic advantage is that the organosilanes used here also catalyze silane and silica condensation. There is thus no need for additional catalysts that must to be removed after synthesis.

## Experimental

**Preparation of the organosilica monoliths.** Silica monoliths were made via sol–gel reaction from bis(3-(trimethoxysilyl)propyl)amine (BTMSPA) and tetramethylorthosilicate (TMOS) or methyltrimethoxysilane (MTMS). Reactions were made such that the total silicon concentration in the reaction mixture was always 16.5 mmol (from TMOS or MTMS and BTMSPA combined; note that BTMSPA contributes two moles of silicon for every mole of silane). The amount of water was 82.5 mmol (1.485 mL) and the amount of acetone or methanol used as solvents was 200 mmol (10.8 mL or 6.4 mL, respectively).

In a typical experiment, TMOS (0.488 mL), BTMSPA (2.254 mL), and acetone (10.8 mL), were mixed in a 50 mL plastic tube. All reactants are liquid and fully miscible at room temperature. The reaction vessel was held in a cooling bath (acetone/dry ice) while adding the components to the mixture to minimize gelation during initial mixing. After mixing, the mixture was allowed to warm to room temperature. Subsequently, water was added. After vigorous shaking, the clear solution was poured into a polypropylene mold, sealed with a septum, and covered with parafilm. After 5 min gelation occurred but all samples were allowed 24 h for further condensation. The resulting material was washed extensively by Soxhlet extraction with methanol and was stored in methanol until further use. When acetone was used as solvent, opaque monoliths were obtained whereas transparent monoliths were obtained when using methanol during synthesis.

**Sample nomenclature.** Organosilicas made from TMOS and BTMSPA are denoted TBMxx and TBAxx, where xx (fraction of silicon provided by monosilane precursor, not from BTMSPA) = 20, 40, 50, 60. “A” denotes samples made in acetone and “M” denotes samples made in methanol. Samples made from MTMS and BTMSPA are denoted MBMxx and MBAxx using the same assignments as above. For example, TBM20 is a sample made with TMOS and BTMSPA in methanol where 20% of the silicon atoms are provided by TMOS and 80% by BTMSPA.

**Preparation of 1-methyl-3-butylimidazoliumsulfonate** [25]. 1-Methylimidazole (40 mmol, 3.28 g) and 1,4-butanesultone (40 mmol, 5.45 g) were mixed in a 50 mL round bottom flask. The mixture was heated to 60 °C for 1 h with stirring under nitrogen. Precipitation of the white zwitterionic salt 1-methyl-3-butylimidazoliumsulfonate (BmimSO_3_) starts after ca. 30 min. As the viscosity of the reaction mixture increases during the synthesis, 10 mL of acetone were added after 30–40 min and the resulting solution was refluxed for 8 h. After filtration, the salt was washed with acetone to remove residues of the starting materials. The white solid was dried overnight at room temperature. C_8_H_14_N_2_SO_3_ (*M* = 218.27 g/mol); ^1^H NMR (300 MHz, D_2_O, δ in ppm): 1.637 (q, 2H); 1.922 (q, 2H); 2.839 (t, 2H); 3.787 (s, 3H); 4.144 (t, 2H); 7.335 (d, 1H); 7.396 (d, 1H); 8.635 (s, 1H); elemental analysis, found (calculated, %): C 44.02 (43.92); H 6.46 (6.31); N 12.83 (12.78); S 14.69 (14.91).

**Preparation of 1-methyl-3-(3-sulfobutyl)imidazolium *para*-toluenesulfonate [BmimSO_3_H][PTS]** [25]. BmimSO_3_ was mixed with *para*-toluenesulfonic acid (p-TSA) in stoichiometric amounts. The mixture was heated to 60 °C and stirred for 4 h to yield 1-methyl-3-(3-sulfobutyl)imidazolium *para*-toluenesulfonate, [BmimSO_3_H][PTS]. C_15_H_22_N_2_S_2_O_6_ (*M* = 390.47 g/mol); ^1^H NMR (300 MHz, D_2_O, δ): 1.577 (q, 2H); 1.843 (q, 2H); 2.234 (s, 3H); 2.782 (t, 2H); 3.712 (s, 3H); 4.056 (t, 2H); 7.252 (ar, 4H); 7.515 (d, 2H); 8.540 (s, 1H). Elemental analysis: found (calculated, %): C 46.14 (44.18); H 5.68 (5.33); N 7.17 (6.93); S 16.42 (15.72). The reason for the deviations between experimental and calculated values is likely due to a slight mismatch between BmimSO_3_ and p-TSA from weighing in the starting materials. EA data did vary slightly from batch to batch, which confirms this hypothesis. Excess BmimSO_3_ or p-TSA can however not be removed.

**Ionogel preparation.** Silica monoliths filled with methanol (from Soxhlet extraction) were placed in a methanolic solution of [BmimSO_3_H][PTS]. After 12 h, the silica monolith was removed from the methanol/IL mixture and placed in the pure IL at 50 °C in a vacuum oven to remove the residual methanol. After 24 h, the methanol was removed from the ionogel as the vacuum had reached a constant value.

**Ionogel nomenclature.** IGs are denoted TBMxxIL, TBAxxIL, MBMxxIL, and MBAxxIL, respectively. For details of organosilica nomenclature see above.

**Spectroscopy.** Infrared (IR) spectra were recorded using the KBr pellet method or the attenuated total reflection (ATR) mode on a Thermo Nicolet FT-IR Nexus 470 with ATR probe head. Spectra were taken from 500 to 4000 cm^−1^ with a resolution of 2 cm^−1^ and 32 scans per measurement.

**Elemental analysis (EA).** EA was done on an Elementar Vario EL III elemental analyzer.

**Thermal analysis.** Simultaneous thermogravimetric analysis-differential thermal analysis (TGA-DTA) experiments were done on a Linseis L81 thermal balance and on a Linseis STA PT-1600 thermal balance in air from 20 to 900 °C with a heating rate of 10 °C/min. Differential scanning calorimetry (DSC) measurements were done on a Netzsch DSC 204. DSC traces were recorded from −150 to 200 °C using liquid nitrogen cooling and a heating rate of 10 °C/min. Isothermal times were 5 min. Samples of ca. 5 mg were placed in aluminum pans with pierced lids. To remove traces of water the samples were heated to 120 °C before the first cooling cycle. Heating and cooling cycles were repeated three times for reproducibility.

**Pore analysis.** Nitrogen sorption experiments were carried out at 77 K on a Quantachrome Autosorb-1. Prior to measurements the samples were degassed in vacuum overnight at 80 °C. Surface areas were calculated via the Brunauer–Emmett–Teller (BET) method [34]. Determination of average pore diameters was done using the Barrett–Joyner–Halenda (BJH) approach using the desorption branch of the sorption isotherms [35].

**X-ray scattering.** Small angle X-ray scattering (SAXS) intensities were recorded at room temperature with a Nonius rotating anode instrument (4 kW, Cu Kα) with pinhole collimation and a MARCCD detector (pixel size: 79 μm). The distance between sample and detector was 74 cm, covering a range of the scattering vector *s* = 2/λ sin θ = 0.04–0.7 nm^−1^ (θ = scattering angle, λ = 0.154 nm). 2D diffraction patterns were transformed into 1D radial averages of the scattering intensity [36]. The pore sizes of the monoliths were evaluated via SAXS using the Porod approach [37–40] with data obtained from BJH pore analysis [35].

The porosity φ of the samples was calculated from BJH data via Equation 1

[1]
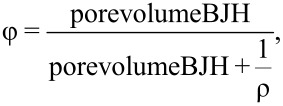


where ρ is the density of the silica, 2.1 g/cm.

**Electron microscopy.** Transmission electron microscopy (TEM) was done on a Philips CM100 electron microscope operated at 80 kV. TEM copper grids were coated with a carbon layer. Samples were ground in an agate mortar and suspended in acetone. One drop of the suspension was deposited on the grid and dried before microscopy. Scanning electron microscopy (SEM) was done on an FEI Phenom desktop electron microscope operated at 5 kV and on a JEOL JSM-6510 with a tungsten filament operated at 15 kV. Energy dispersive X-ray spectroscopy (EDXS) was done with an Oxford Instrument INCAx-act X-ray detector. Prior to measurements the samples were coated with a 100 nm carbon layer using a POLARON CC7650 Carbon Coater.

**Electrochemical impedance spectroscopy (EIS).** For EIS the dry IGs were contacted with a graphite paper layer and sandwiched between platinum electrodes. The graphite paper layer was used as a sacrificial layer (used one time for one sample) to avoid a direct contact with the platinum electrode. Even though the IGs are rather stable, their surfaces may be slightly sticky; this especially applies at higher temperatures. The roughness between the graphite and ionogel is average as observed by the angle of the Nyquist curve with the *x*-axis in the low frequency domain. The angle is about π/4, which is typically observed for such contact electrodes (the absence of any roughness will result in an angle of π/2). The variation of this angle does not modify the characteristics of the samples, i.e., the conductivity and the dielectric behavior as given by the intercept of the curve with the *x*-axis and in the high frequency domain, respectively.

Conductivity measurements were performed by the complex impedance method carried out with a Solartron 1174 frequency analyzer. The frequency range was from 1 to 10^6^ Hz and the temperature cycle was between 258 and 473 K as a cooling/heating/cooling sequence. The customary model using constant phase elements was applied to simulate the impedance spectra. After removal of the geometric capacitance of the cell, the impedance plots were refined by using CPE_p_ and CPE_s_ constant phase angle elements, which are related to the dielectric relaxation of the material and electrode phenomena, respectively. Their related impedance is expressed as CPE_p_ = K^−1^(*j*ω)^−n^ and CPE_s_ = Q^−1^(*j*ω)^−p^ (0 ≤ (n, p) ≤ 1).

**Figure 1:**
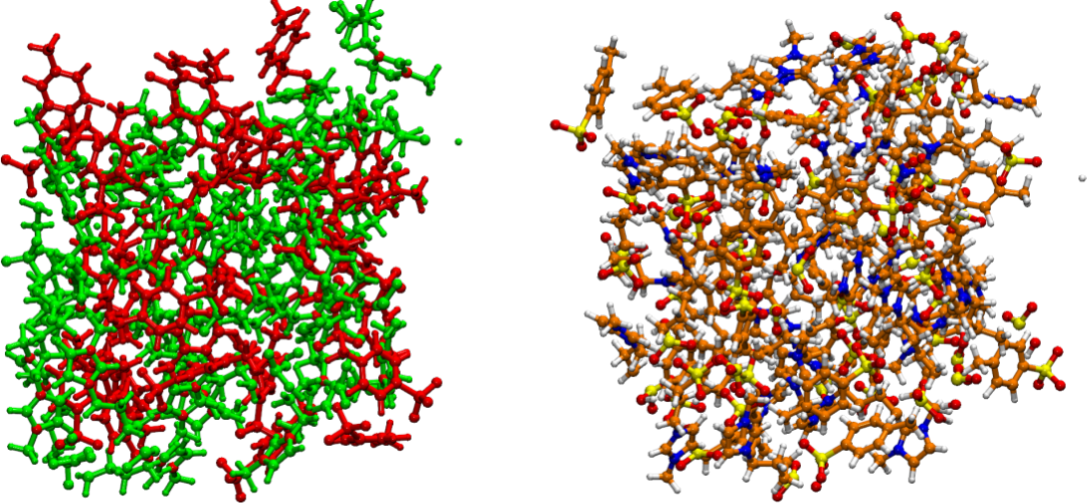
Ball-and-stick representation of the simulation box. Visualized with TRAVIS [43] and vmd [44]. Left: cations in green and anions in red. Right: carbon in orange, sulfur in yellow, oxygen in red, nitrogen in blue, and hydrogen in white.

**Computational methodology.** The ab initio molecular dynamics (AIMD) simulations in which the forces are calculated from the electronic structure on the fly were carried out as described previously [41] using the cp2k program packages [42]. AIMD simulation was started from a classical molecular dynamics simulation snapshot of 32 ion pairs of [BmimSO_3_H][PTS] under periodic boundary conditions. The snapshot can be obtained upon request. In the classical MD simulation the system was set up for a density of 1.6 g/cm^3^ and NPT simulations were performed resulting in a box size of 2349.41 pm^3^. The AIMD simulations we run at 440 K with massive thermostats (parameters as in [41]) for 5 ps. After this the simulation was done in an NVT ensemble to provide 2.5 ps production trajectory at 450 K which was analyzed with the software TRAVIS [43]. A forthcoming publication will provide more details and longer simulation times. Figure 1 shows the simulation box used for the experiments.

## Results

### Silica monoliths

As stated in the introduction, mesoporous silica monoliths are often brittle and thus difficult to handle. The first goal of the current study is thus to provide a synthetic protocol towards more robust silica matrix materials suitable for IL incorporation. The second goal is to produce IGs with high proton conductivities.

**Figure 2:**
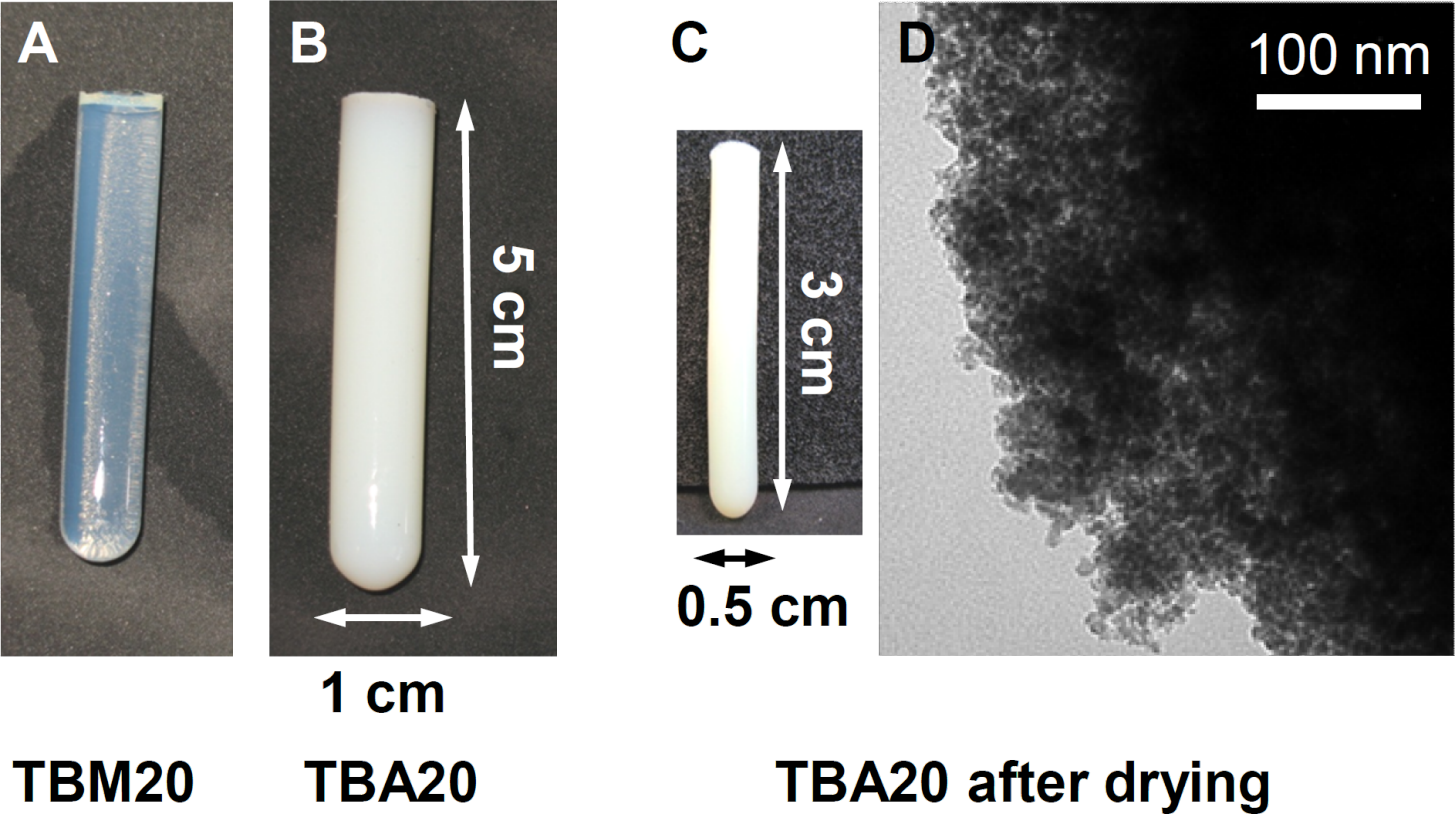
Representative photographs and TEM images of organosilica monoliths. (A) Wet TBM20 after Soxhlet extraction with methanol, (B) wet TBA20 after Soxhlet extraction with methanol, (C) dry TBA20 after drying at ambient conditions, (D) TEM image of TBA20 after drying.

One viable strategy to obtain mechanically robust silica hosts is the use of organic linkers to provide additional flexibility and stability to the silica network [45–46]. The current study therefore focuses on organosilica monoliths with varying ratios of the silicon alkoxide precursors TMOS and BTMSPA ranging from 20:80, 40:60, 50:50, to 60:40 in two different solvents, methanol and acetone. All monoliths remain intact after Soxhlet extraction, Figure 2. After drying at ambient conditions, the monoliths shrink but again remain intact. The monoliths synthesized in methanol are clear to translucent as long as they are wet, but turn opaque upon drying. The monoliths grown in acetone are opaque already after the sol–gel reaction and Soxhlet extraction.

Transmission electron microscopy (TEM, Figure 2) images obtained from ground samples show that the size of the primary silica particles making up the monoliths is around 10 nm. TEM also shows that the materials do not have a periodic order, but contain disordered pores. This observation applies to all organosilica monoliths, regardless of the solvent, the precursors, or the precursor ratios used during synthesis.

**Figure 3:**
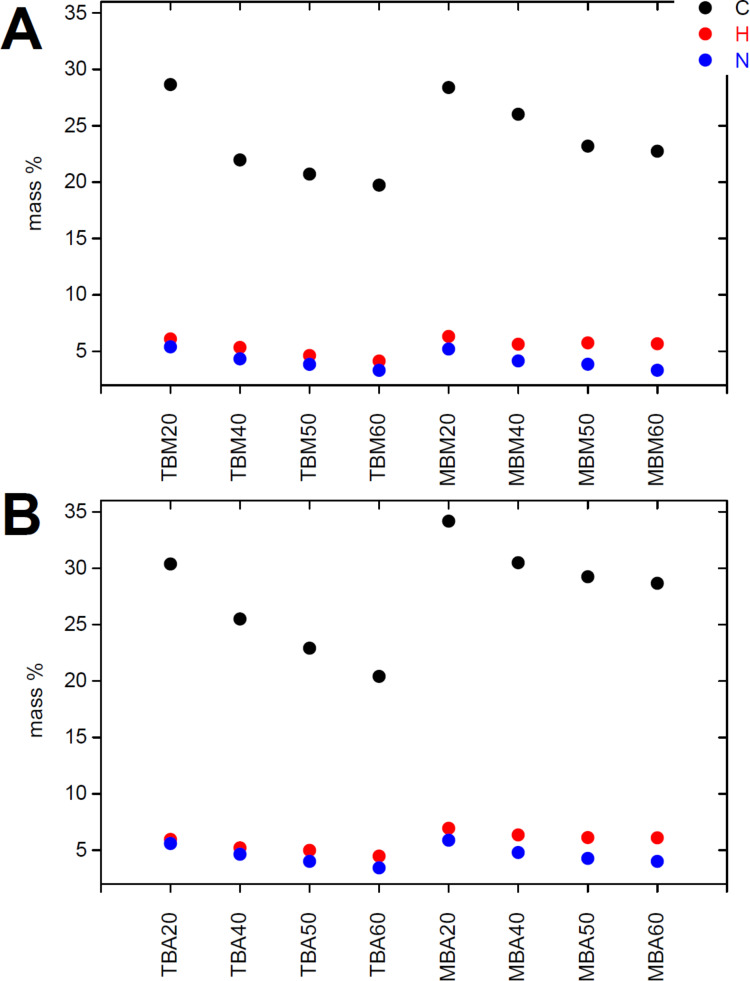
Carbon, hydrogen, and nitrogen contents of silica monoliths made from methanol (A) and acetone (B).

Figure 3 shows elemental analysis (EA) data of all organosilica monoliths. Samples made with MTMS have slightly higher carbon and hydrogen contents than the samples made with TEOS. This is partly due to the additional methyl group present in MTMS, possibly also to a slightly higher rate of MTMS incorporation. Generally the content of carbon, hydrogen, and nitrogen decreases as the organosilane fraction (MTMS or BTMPSA, respectively) decreases and the fraction of TMOS (which does not introduce organic moieties) increases. Comparison between samples of the same composition but made in acetone or methanol only shows minor differences, which are within the sample to sample variation typically observed in these reactions.

**Figure 4:**
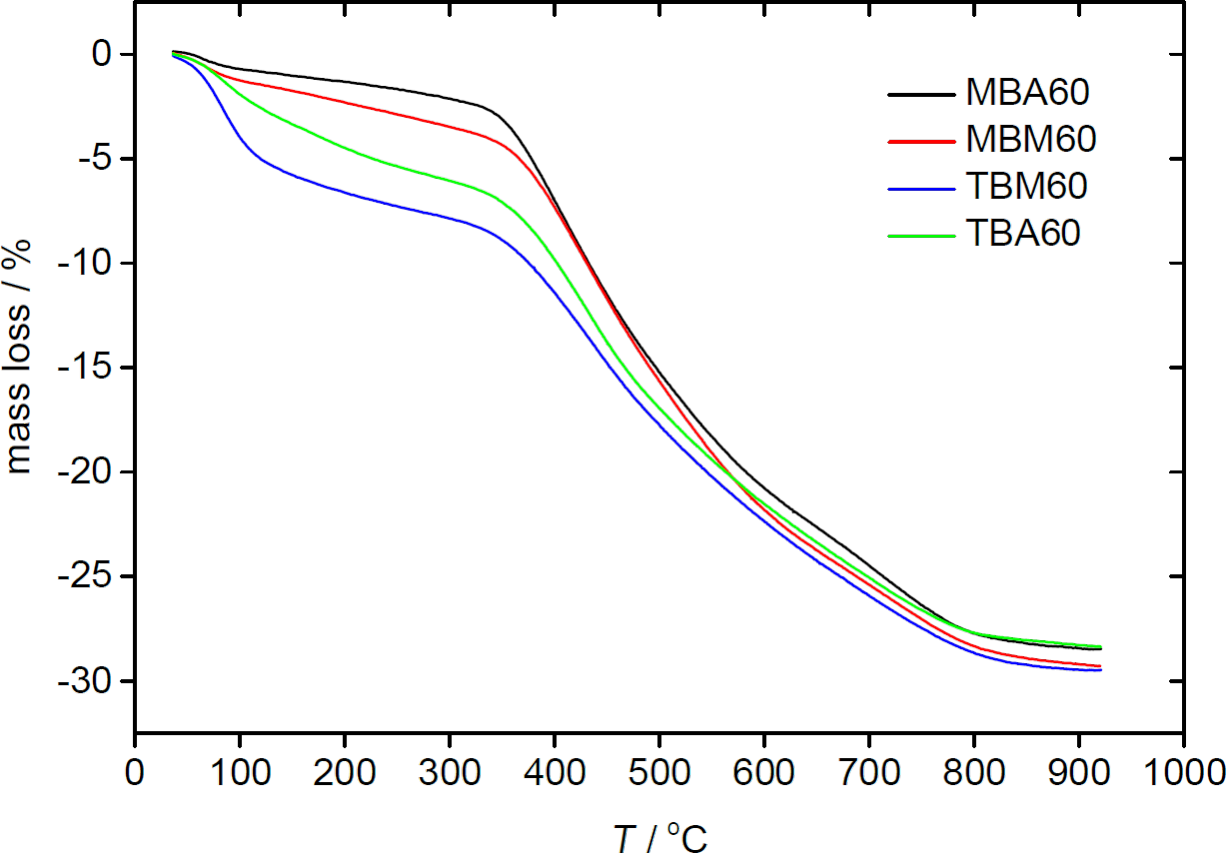
TGA data of pure organosilica monoliths.

Figure 4 shows representative thermogravimetric analysis (TGA) data of the pure organosilica host materials. All TGA curves exhibit a three-step degradation process. The first step between 35 and ca. 120 °C is due to the loss of surface-bound water. Generally, the monoliths made from TMOS (TBA60 and TBM60) show higher weight losses (up to ca. 5%) in this region than samples made from MTMS, which only show a loss of up to 1.5%. This difference is attributed to the methyl group of MTMS possibly rendering the material slightly more hydrophobic and thus reduces water uptake.

The second weight loss between ca. 120 and 300 °C is assigned to condensation of residual silanol groups and further water evaporation. Possibly this is accompanied by elimination of some organic fragments from the organic linkers. The last weight loss from 300 to ca. 870 °C is largely due to the oxidative elimination of the organic linker [47] and further condensation of the silanol groups. The remaining weight at 900 °C is due to silica and carbon residues.

**Figure 5:**
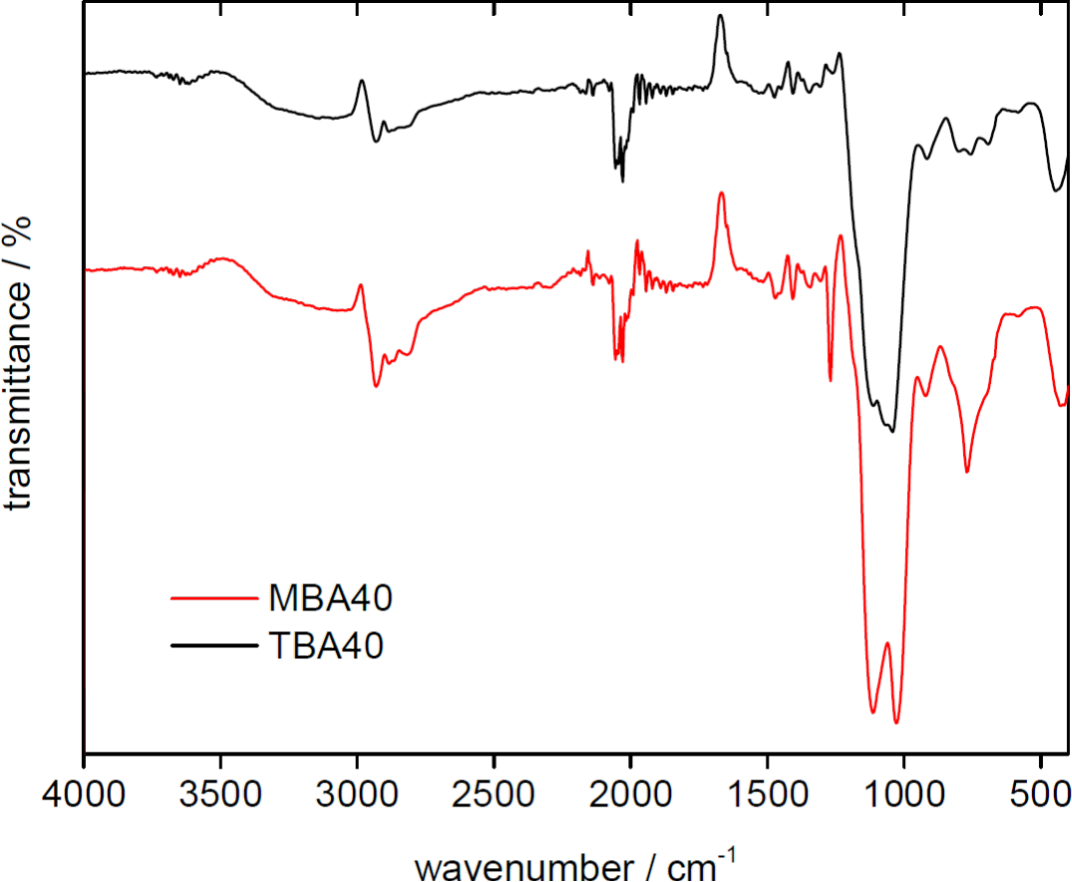
Representative IR spectra of TBA40 and MBA40.

Figure 5 shows representative ATR-IR spectra of two dried monoliths. The spectra of all samples are virtually identical and exhibit fairly broad bands in all cases. All spectra show C–H bending vibrations at 770, 850, and 950 cm^−1^. Bands at 900, 1010, and 1080 cm^−1^ are due to asymmetric and symmetric Si–O–Si stretching vibrations and Si–OH silanol groups on the silica surface. Broad bands between 3000 and 3400 cm^−1^ originate from Si–OH, H_2_O, C–N, and N–H stretching vibrations. A further characteristic N–H stretching vibration is observed at 2055 cm^−1^ [48].

**Figure 6:**
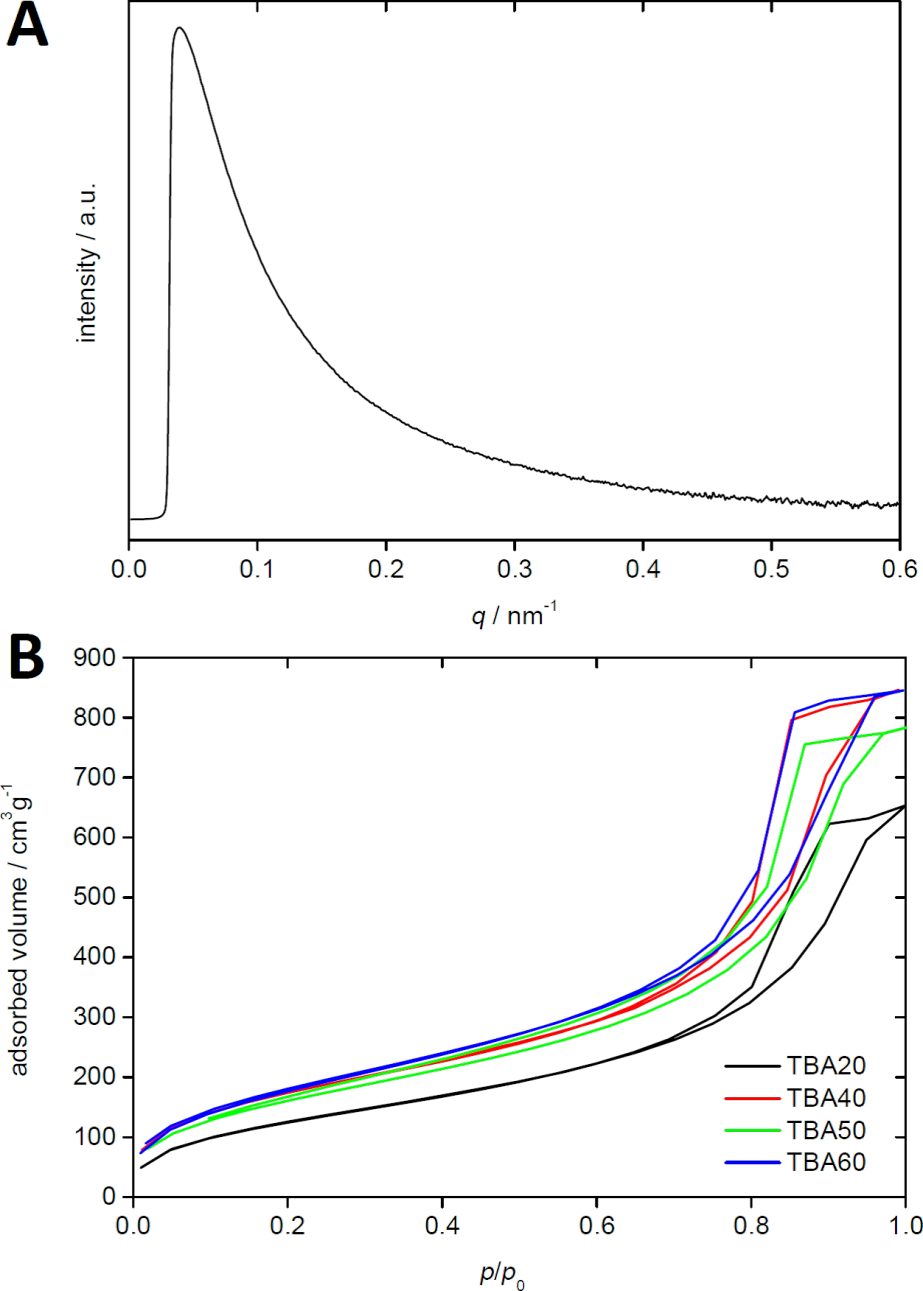
(A) SAXS pattern of TBA40 and (B) nitrogen sorption data of TBA20 to TBA60.

Figure 6 shows a representative small angle X-ray scattering (SAXS) pattern. All SAXS patterns show a strong scattering signal with a typical *q*^−4^ behavior indicating an amorphous, mesoporous system with strong phase boundaries and cylindrical pores, but without ordered mesopores [37].

This is confirmed by nitrogen sorption measurements, Figure 6. All monoliths show a type IV isotherm typical of mesoporous solids. The H2 hysteresis loop is due to capillary condensation in the mesopores and is attributed to differences between condensation and evaporation processes occurring in pores with narrow necks and wide cavities often referred to as ink bottle pores. The late onset of significant nitrogen uptake and the parallel adsorption and desorption branches also indicate the presence of rather small pores and a relatively narrow pore size distribution [37]. Analysis of the SAXS data using the Porod-approach [37–40] confirms this, although pore sizes determined via SAXS are slightly larger than the values determined from BJH analysis, Table 1.

**Table 1:** Surface areas, pore volumes, porosity, and BJH average pore sizes derived from SAXS and nitrogen sorption (BET).

sample	BET surface area [m^2^/g]	BJH pore volume [cm^3^/g]	Open pore volume [%]	average pore size radius (BJH) [nm]	average pore size radius (SAXS) [nm]

TBA20	466	0.95	66.5	6.3	8.3
TBA40	634	1.23	72.2	6.2	9.5
TBA50	523	1.14	70.5	6.9	8.2
TBA60	518	1.31	73.4	6.4	8.0
TBM20	506	0.35	42.4	1.9	3.7
TBM40	350	0.19	28.5	1.9	2.5
TBM50	309	0.18	27.4	1.9	2.4
TBM60	752	0.82	63.4	2.1	7.8
MBA20	478	0.66	58.2	3.8	6.2
MBA40	710	1.00	67.8	4.9	8.2
MBA50	746	0.69	59.2	3.2	6.4
MBA60	740	1.14	70.6	5.0	13.5
MBM20	188	0.18	27.8	1.8	3.4
MBM40	383	0.23	32.8	1.9	3.4
MBM50	248	0.17	26.8	1.9	2.9
MBM60	363	0.29	37.7	1.2	3.7

Table 1 summarizes the data obtained from SAXS and nitrogen sorption. All organosilica materials have surface areas between 250 and 750 m^2^. In general, silica materials made from acetone have larger surface areas than the materials made from methanol. The surface areas are remarkably high for monolithic silica materials dried under ambient conditions (xerogels) [49–50]. Moreover, many mesoporous systems collapse because of the strong forces applied to the pore walls by the evaporating solvent [49]; this is, however, not the case here. We never observe cracking or other pore collapse. This is likely due to the flexibility of the organic bridges that enable the dry solid to respond to local mechanical stress without breaking or pore collapse.

Table 1 also shows that the pore size and open pore volume strongly depend on the composition of the monolith and the solvent. Monoliths made in methanol have much smaller pore radii (ca. 2 nm) than silica monoliths made in acetone (4 to 6 nm). If made in methanol, the surface area depends on the amount of TMOS or MTMS. TMOS results in surface areas that are about 200 m^2^/g higher than surface areas of samples made from MTMS. Overall the open pore volume of these samples is similar and around 30–40%. Moreover, monoliths made from acetone generally have much higher surface areas of 500–750 m^2^ than monoliths made from methanol. The synthesis protocol using MTMS and acetone results in the highest surface areas.

Overall, organosilica monoliths prepared with TMOS in acetone shows the highest open pore volumes, the largest pores with 6 to 7 nm in radius, and the highest pore volumes of up to 1.31 cm^3^/g. An increasing amount of the linker BTMSPA decreases the pore volume, pore size, and open pore volume. The same observation applies to materials obtained with MTMS instead of TMOS in acetone. High surface areas up to 740 cm^3^/g, high open pore volumes of around 60%, and pore sizes between 3 and 5 nm are also obtained in this system.

In contrast, the methanol-based synthesis yields no clear trend in the product characteristics, except for the fact that the pore radius is around 2 nm in all samples. The precursor TMOS results in slightly higher surface areas compared to MTMS. Overall the pore volume are much smaller than those for acetone-based silica.

### Ionogels

As stated in the introduction, the goal of the current study is the evaluation of new IGs for proton transport. The following section thus presents the results of IL synthesis and the properties of the IGs resulting from the combination of the IL with the different organosilica host materials.

**Scheme 1:**
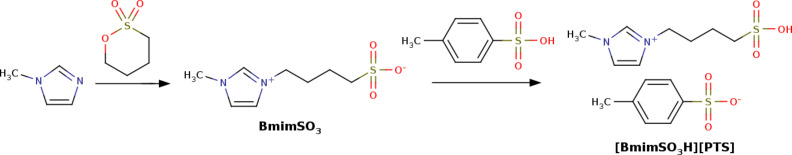
Synthesis of the IL [BmimSO_3_H][PTS].

The synthetic protocol (Scheme 1) is adapted from our previous study [25]. However, as 1,3-propanesultone (which was used in the previous work) has a series of safety issues (see the corresponding MSDS) that make it unattractive for larger scale or commercial application, we have replaced 1,3-propanesultone with 1,4-butanesultone for the current work. Accordingly, the new IL used in this study is 1-methyl-3-(3-sulfobutyl)imidazolium *para*-toluenesulfonate, [BmimSO_3_H][PTS]. [BmimSO_3_H][PTS] carries two sulfonate groups, one on the sulfobutyl side chain of the imidazolium ring and one in the *para*-toluenesulfonate anion. These two sulfonate groups share a proton and are thus expected to enhance proton conductivity, identical to the previous example [25].

**Figure 7:**
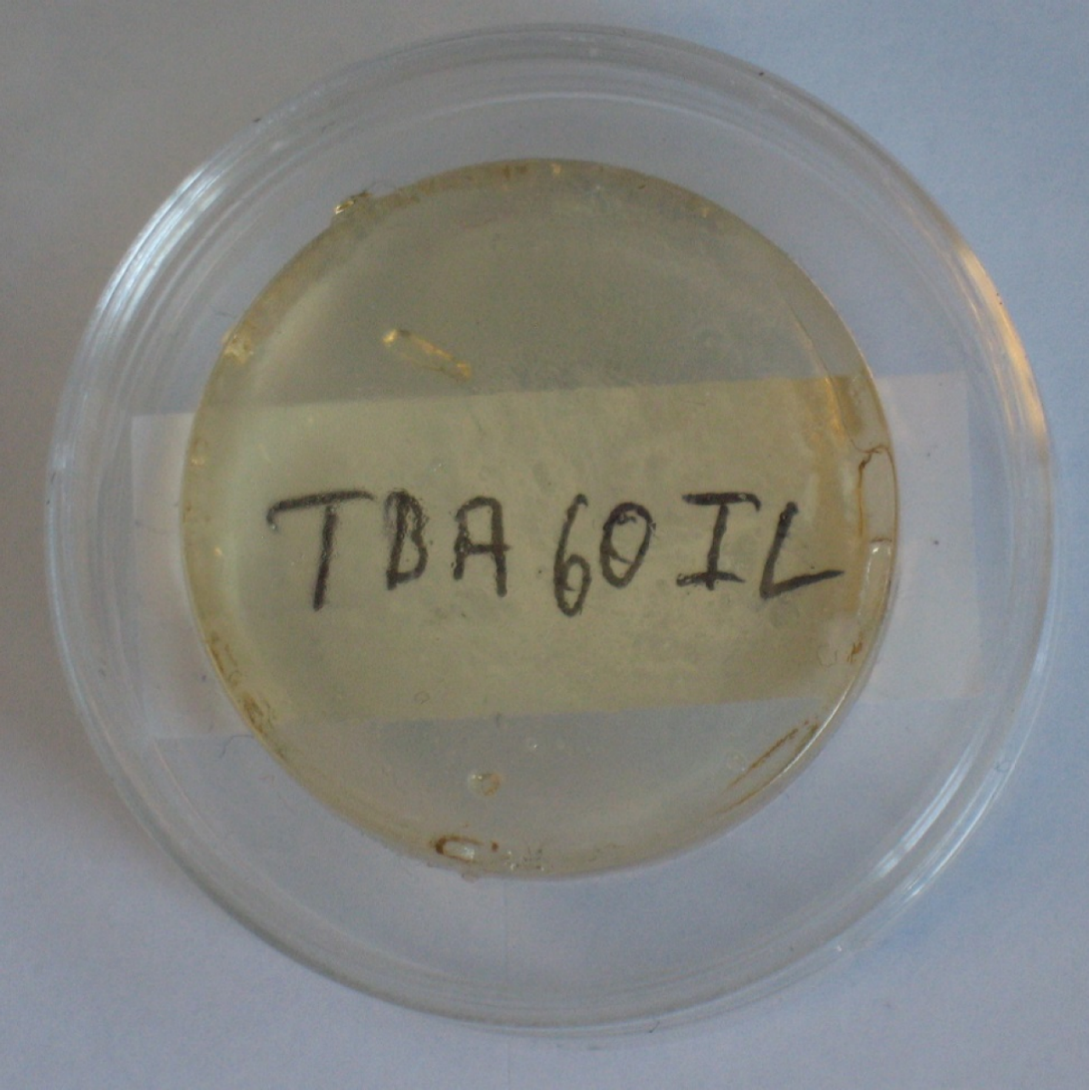
Photograph of an IG resulting from the combination of the TBA60 organosilica matrix and [BmimSO_3_H][PTS].

The combination of [BmimSO_3_H][PTS] with the organosilicas described above yields IGs with the shapes and dimensions of the respective organosilica matrix. In touching the materials they exhibit a rubberlike consistency, but this aspect has not been evaluated here. Figure 7 shows that the IGs are clear and translucent materials. In some cases, they are slightly yellow; the reason for this color is not quite clear because both the neat IL and the monoliths before IG formation are almost colorless. Some monoliths are, however, somewhat off-white or turbid. This could contribute to the slight color change and is mostly observed for IGs obtained via the acetone-based synthesis (TBA and MBA materials).

The IL is tightly trapped in the organosilica matrix and does not show signs of leakage when stored at ambient conditions over several months. Moreover, the IGs do not show degradation (such as color changes or crack formation) even when stored in a vacuum oven at 250 °C for several days.

**Figure 8:**
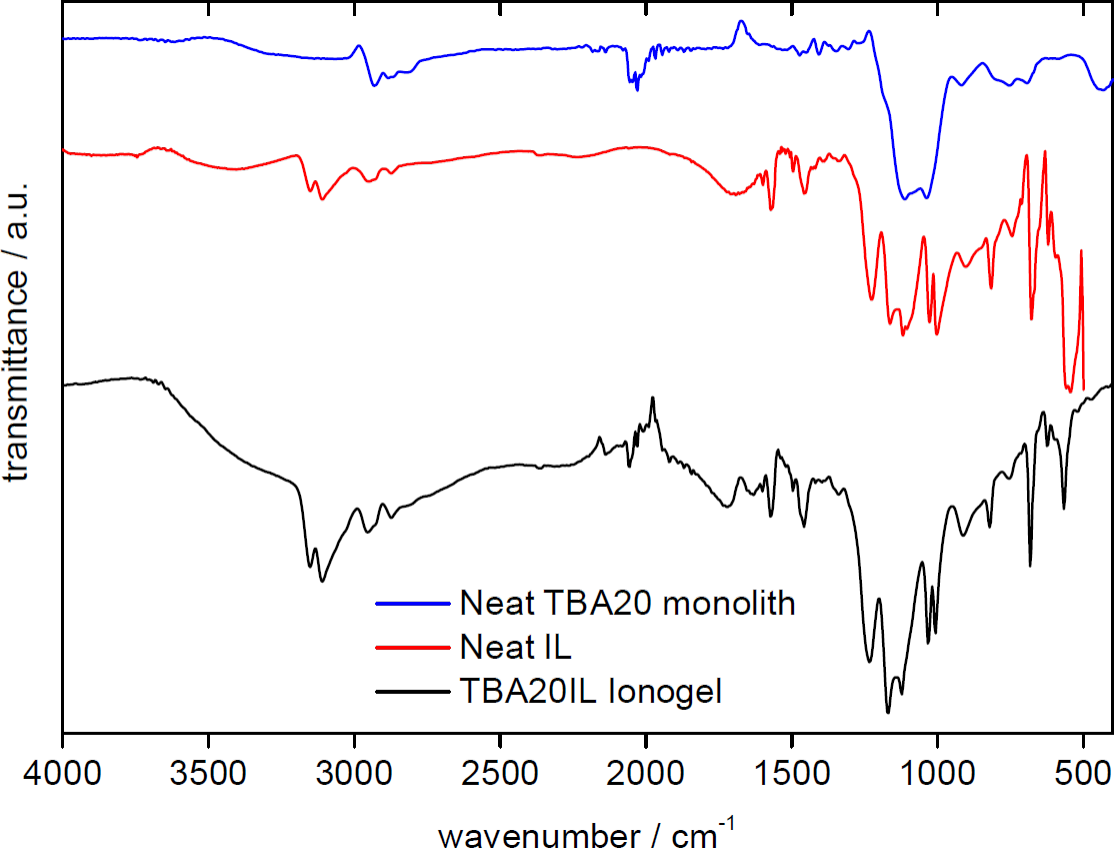
IR spectra of a neat TBA20 monolith, pure IL, and the resulting TBA20IL IG.

Figure 8 shows representative IR spectra of a neat TBA20 monolith, the neat IL, and the TBA20IL ionogel resulting from the combination of the two components. The distinct bands of the IL dominate the IG spectrum and indicate that the IL is the major component of the IG. Bands around 2900 and 2000 cm^−1^ also show the presence of the silica material.

In detail, in the spectra of the neat IL [BmimSO_3_H][PTS], the bands can be assigned to the C–N and C–H stretching vibration modes of the imidazolium ring (3151, 3112, 2957, and 2922 cm^−1^), CH_3_–N and CH_2_–N stretching modes and C–C stretching vibrations of the imidazolium ring (1602, 1573, 1456, and 1229 cm^−1^), and out of plane C–H bending vibrations (816, 743, and 681 cm^−1^). The two characteristic bands at 1028 and 1116 cm^−1^ can be attributed to the asymmetric and symmetric stretching vibrations of the sulfonate group [48]. Between 500 and 1700 cm^−1^ the spectra of the neat IL and the IGs are virtually identical indicating a dominant contribution of the IL to the IR spectra.

**Figure 9:**
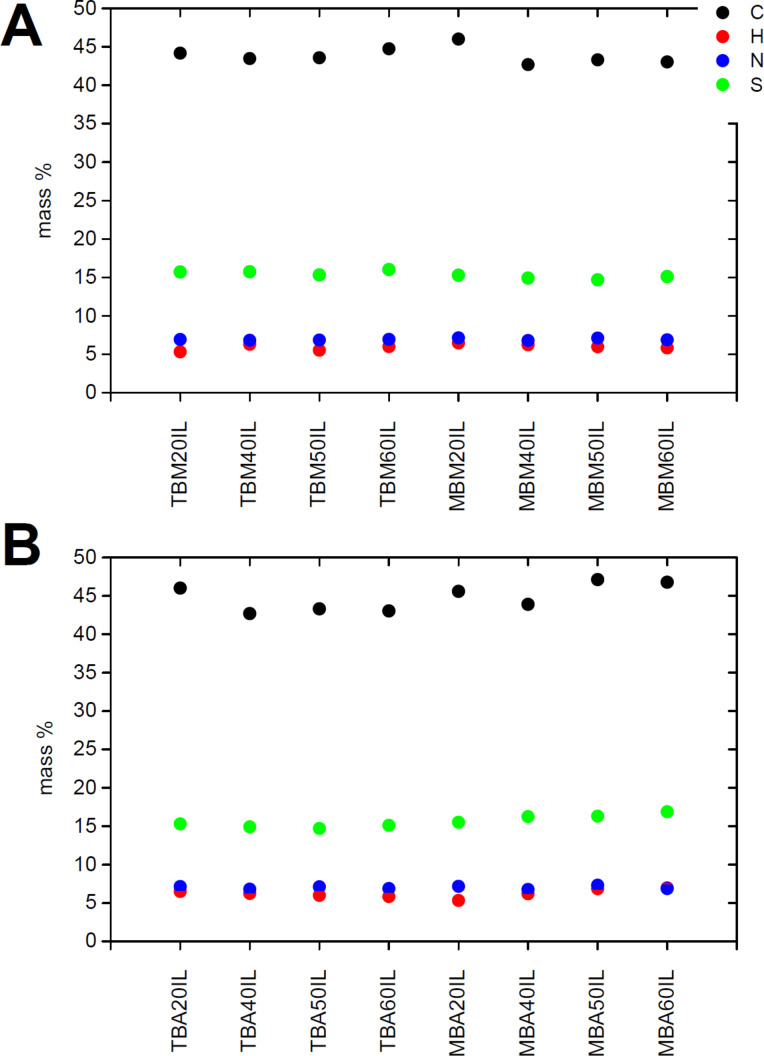
EA data of IGs. Samples shown in panel (A) were made via the methanol-based synthesis and samples shown in panel (B) were made via the acetone-based synthesis of the organosilica. It must be noted here that for reasons described in the Experimental part, there is a slight variation in the IL composition. Moreover, the organisilica composition also slightly varies from batch to batch. As such it is rather difficult to exactly assign compositions from EA data.

Figure 9 shows the results from EA of the IGs. Overall, EA data do not show significant variations between the individual IGs. All IGs based on monoliths made from methanol have carbon contents between 42 and 44%, hydrogen contents between 5 and 7%, nitrogen contents between 6 and 7%, and sulfur contents around 15%. In the case of the IGs based on the monoliths made from acetone, the carbon contents are somewhat more scattered and vary from around 40 to 48%, while the H, N, and S contents are again rather constant at around 5 to 8%, 5 to 8%, and 15 to 17%, respectively.

**Figure 10:**
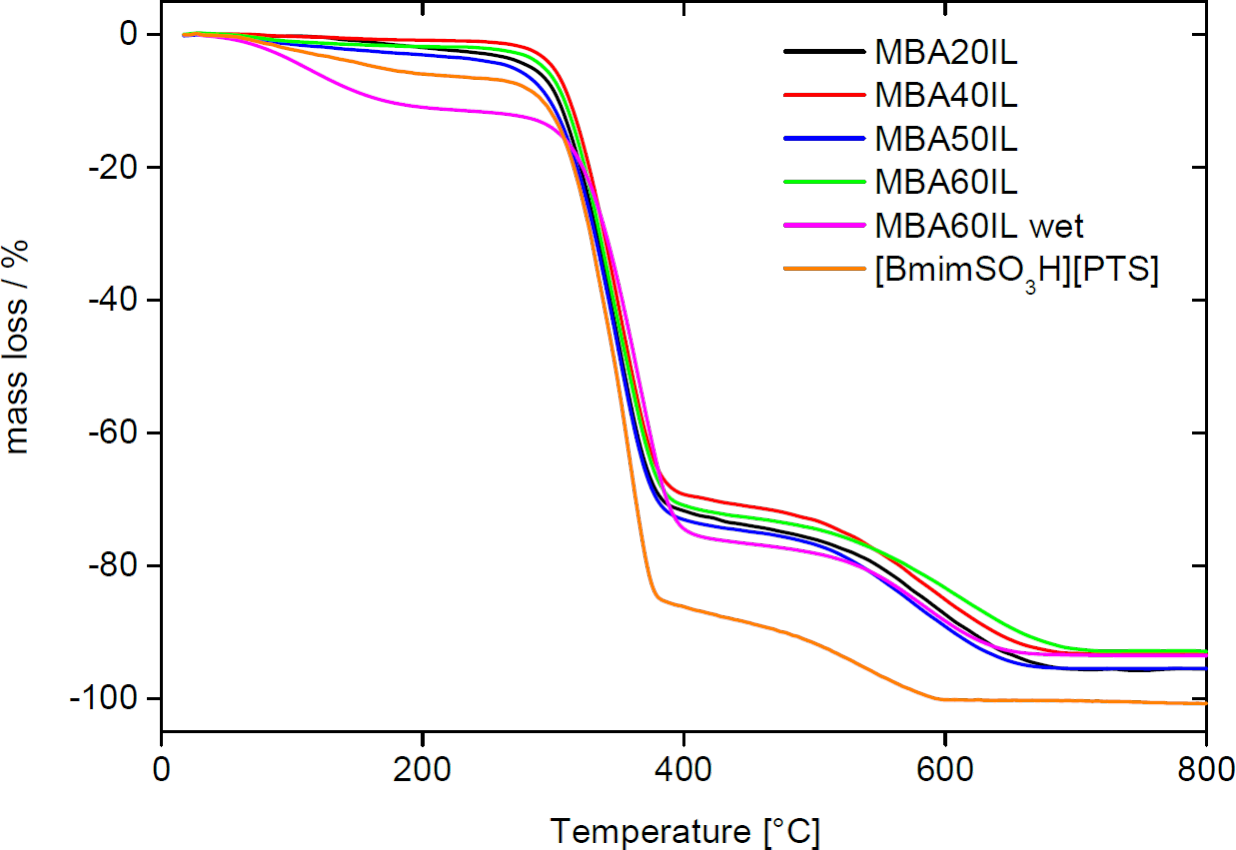
TGA data of MBA20IL, MBA40IL, MBA50IL, and MBA60IL along with TGA trace obtained from wet MBA60IL obtained without drying before TGA, and a TGA trace of the pure IL [BmimSO_3_H][PTS].

Figure 10 shows representative TGA data obtained from the IL and the MBA IGs. If the IGs are dried in vacuum before the TGA experiments, they are stable up to 250 °C with a maximum mass loss of only around 3%. This is presumably due to traces of water in the IL, to condensation of remaining silanol groups, or to traces of organic solvent from the synthesis.

Between 260 and 420 °C a significant mass loss of ca. 70% is observed. It is mainly due to the decomposition of the IL but the decomposition of the organic linker in the organosilica matrix also contributes to this weight loss. The last weight loss of ca. 25% between 500 and 700 °C is assigned to the complete decomposition of the IL and further water release associated with silanol condensation.

Complete decomposition of the neat IL is already achieved at 600 °C. Moreover, a comparison of IGs that were dried before TGA with identical wet IGs directly from the synthesis shows that the overall water uptake of the IG can reach ca. 12% when stored at ambient conditions rather than under vacuum. Even when dried, the IL alone already contains ca. 6% of water. Overall, TGA thus shows that the IGs consist of around 85 to 90% of IL by weight (not considering the contribution of the organic linker moiety in the organosilica host) and 10 to 15% of organosilica matrix.

**Figure 11:**
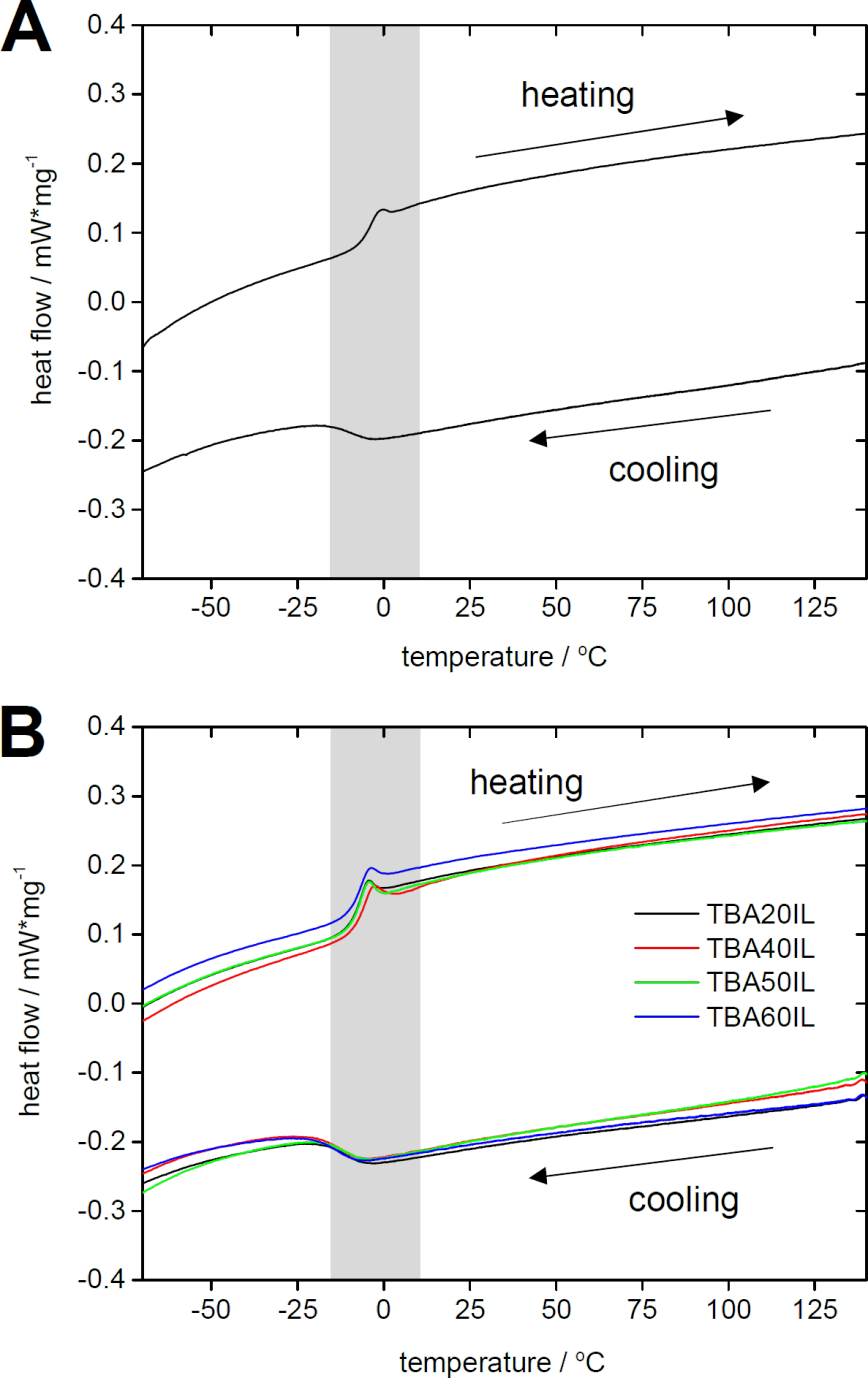
DSC heating traces of (A) [BmimSO_3_H][PTS] and (B) the resulting TBA IGs. Gray areas highlight the region of the glass transition.

Figure 11 shows representative DSC traces of the neat IL and the samples TBA20IL to TBA60IL. The neat IL (grey line) exhibits a glass transition at *T*_g_ = −11.9 °C. No crystallization or melting peaks are visible in the range of −170 to 150 °C. The fact that the glass transition is less visible in the cooling curves is due to supercooling and indicates rather slow dynamics of the IL due to high viscosity, similar to our previous example [25]. Table 2 shows that the glass transition temperatures do not change significantly by entrapping the IL inside the mesoporous silica networks made from acetone. The variations of *T*_g_ in the host materials made in methanol are somewhat more pronounced but also here *T*_g_ remains at around −9 °C.

**Table 2:** *T*_g_ values obtained from DSC measurements (second heating cycle). The *T*_g_ of the pure IL [BmimSO_3_H][PTS] is −11.9 °C. n.o. = not observed.

	*T*_g_ [°C]			
	
	TBA	TBM	MBA	MBM

20	−9.8	−10.0	−9.7	−10.4
40	−9.1	−6.2	−10.0	−9.0
50	−9.6	−8.1	−10.2	−8.4
60	−9.9	−9.4	n.o.	n.o.

It has been pointed out by a referee of this manuscript that – because the DSC signals are around 0 °C – water may also be involved in these processes. As detailed in the Experimental section however, the samples underwent a first heating/drying cycle to 120 °C and the subsequent cycles were run up to 200 °C, suggesting that the vast majority of the water possibly present in the IL was removed. Moreover, DSC traces obtained from third and fourth heating cycles are identical to those shown in Figure 11, thus confirming this assumption.

**Figure 12:**
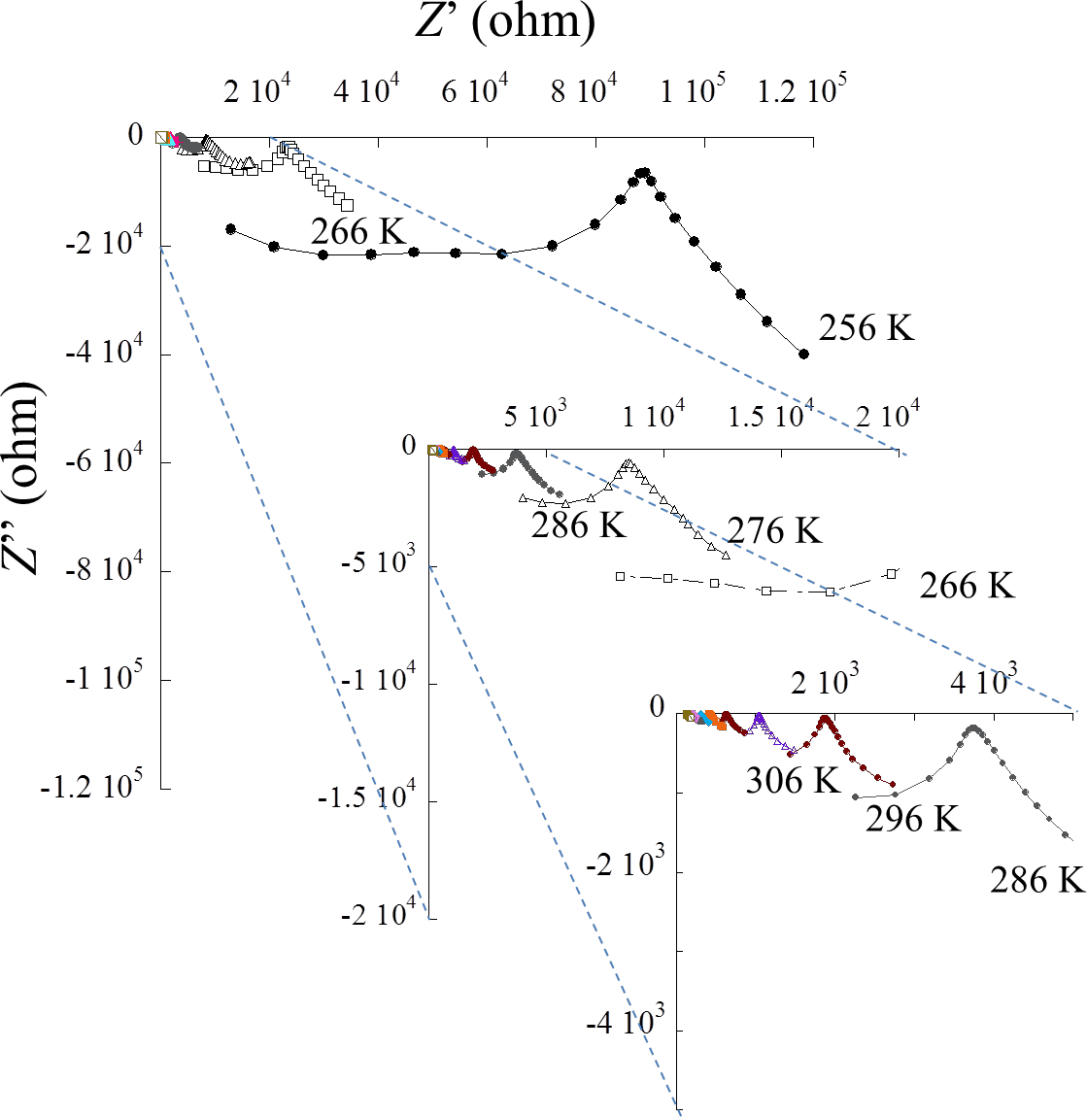
Nyquist curve *Z*”(ω) vs *Z*’(ω) for TBA20IL with zoom-ins to the central areas of the plot vs temperature.

Electrochemical impedance spectroscopy (EIS) was used to evaluate the electrical behavior of the IGs. Figure 12 shows a representative Nyquist plot of TBA20IL, where the imaginary part of the impedance is described as a function of the real part (*Z*” vs *Z*’) in orthonormal basis. Clearly, the impedance depends on the temperature. This is expected because the ion mobility within the sample is activated by temperature. To a first approximation, the intercept of the depressed semi-circle with the *z*’-axis (or the intercept of the straight line with the *z*’-axis) provides information on the resistance of the material. A temperature increase shifts the intercept to lower *z*’ values indicating a lower resistance and hence a higher conductivity.

Indeed, the general feature changes drastically with the presence of the depressed semi-circle and the straight line at low temperature, then the depressed semi-circle disappears progressively, and the straight line at higher temperatures becomes more pronounced. This behavior corresponds to the freezing of the ion mobility at lower temperature where the semi-circle represents the inside bulk that fades when increasing the temperature until the presence of polarization and diffusion is only visible by the straight line.

More specifically, the lower frequency response visualized as a straight line is representative of the electrode polarization, while the higher frequency response forming the depressed circle is due to the dielectric properties of the bulk. The first part can be described as a non-ideal capacitor (constant phase element) in series with the interfacial resistance while the second part is fitted with a constant phase element in parallel with the same interfacial resistance. The divergence from ideal behavior (Debye case) at high frequency is displayed by the depressed circle and the fact that the circle does not close on the *z*’-axis. The related impedance of the capacitor is given in the Experimental part.

For the lower frequency region, the straight line is not parallel to the *z*”-axis, which should be expected from an ideal capacitor (forming an angle π/2 from *z*’-axis). The deviation is due to the roughness of the interface between the sample and the blocking electrode.

**Figure 13:**
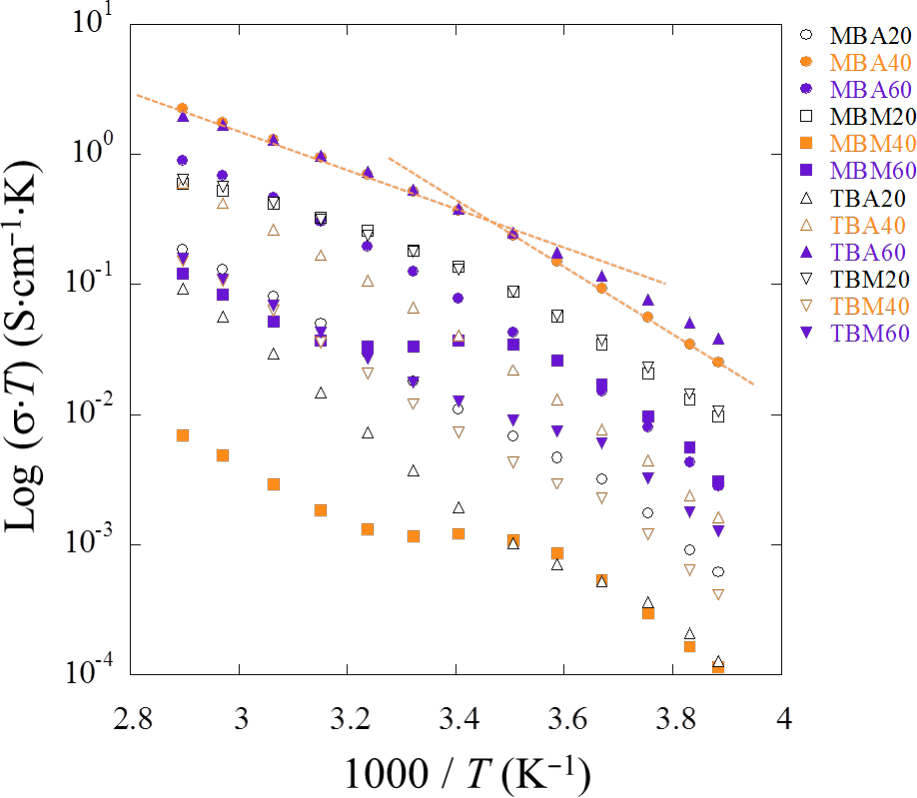
Variation of log(σ·*T*) versus 1000/*T* for all IGs. The change in the energy of activation likely does not stem from a weight loss process such as a weak dehydration because there is no apparent thermal event below 100 °C. However, data collected by DSC clearly show a transition at around −9 °C. This is reasonably close to 12 °C, i.e., 1000/*T* = 3.5 K^−1^, where the change in *E*_a_ occurs. Le Bideau and coworkers performed quasi-elastic neutron scattering experiments to address the presence of a soft transition responsible of the change in the conductive dynamics [52] but as there are no data available for the present system we prefer to not speculate about the details of the kink observed in some of the data. The experiments are, however, highly reproducible.

The components of the so-called equivalent electrical circuit are refined using the entire frequency domain. The fitted resistances as a function of the temperature are used to determine the conductivity and they are displayed log(σ·*T*) as a function of the inverse of the temperature (1000/*T*). The points describe a straight line disrupted at one point (1000/*T* ≈ 3.5 K^−1^, i.e., *T* = 285 K, Figure 13).

Surprisingly, for some compositions, especially MBM40IL, MBM60IL, and TBM60IL, a plateau between both linear regions appears. This intermediate regime, suggesting that the mobility is not thermally activated anymore, can be understood as a transient behavior. These two linear variations rather than a slightly curved feature suggest the presence of a thermally activated Arrhenius-type process rather than a Vogel–Tammann–Fulcher (VTF) type process. To ensure the true occurrence of these two linear variations the derivative, d(log(σ·*T*)/d(1000/*T*), was analyzed. The derivative forms a stair-type feature indicative of two distinct slopes, i.e., two distinct energies of activation. In contrast, a VTF mechanism would produce an inclined straight line on the derivative. Such a behavior is, e.g., observed for ILs in soft matrices such as polymers [14–16,51].

All samples show an Arrhenius-type behavior with two distinct energies of activation. MBA40 (followed by TBM20 and MBM20) performs the best with σ = 7 × 10^−3^ S·cm^−1^ at 357 K and σ = 3.4 × 10^−3^ S·cm^−1^ at 293 K. This is associated with an activation of energy *E*_a_ of 0.6 eV between −20 °C and 20 °C and subsequently with an *E*_a_ of 0.45 eV from 20 °C to 80 °C. It shows that the charge-carrier mobility is less activated with temperature when the temperature increases.

The conductivities obtained from the measurements are comparable to other IGs where the IL confinement leads to an intermediate state between liquid and solid (conductivity vs mechanical strength, respectively), since σ_298 K_ = 3.15 × 10^−3^ S·cm^−1^ and σ_343 K_ = 1.12 × 10^−2^ S·cm^−1^ for one of the best polymer nanocomposite ionogel composed of modified PMMA bearing trimethoxysilane groups and tetraethoxysilane (TEOS) with 76 wt % of 1-butyl-3-methylimidazolium bis(trifluoromethane sulfonyl)imide ([BMIm][NTf2]) [15]. A critical threshold in ionic conductivity around this value of 76% was explained by a transition from a polymer in salt to a quasi-liquid state [14]. It is noteworthy that the IL content in the MBA series is somewhat higher at 85 to 90% as derived from TGA analysis (Figure 10).

**Figure 14:**
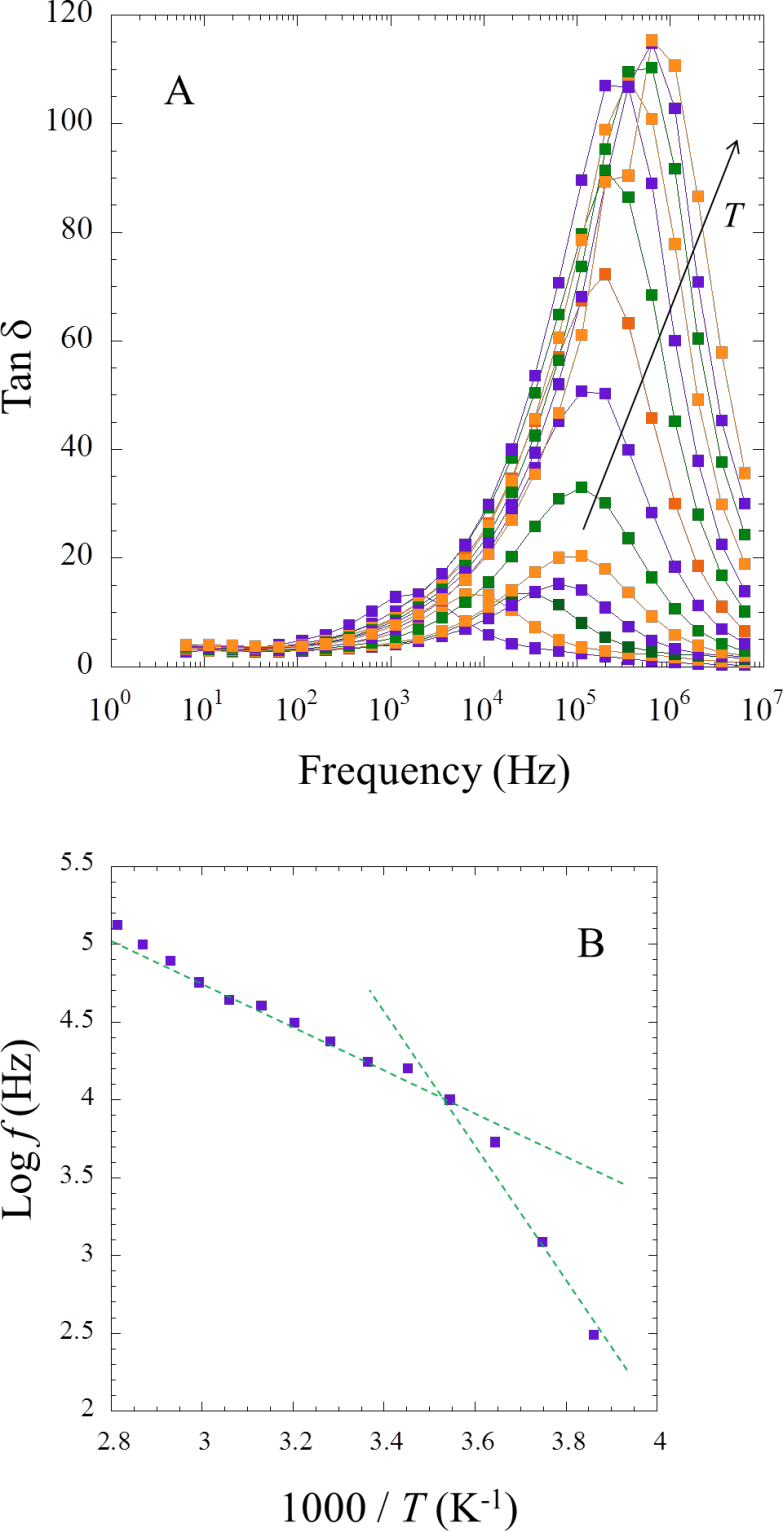
Variation of (A) tan δ vs log *f* and (B) relaxation time vs 1000/*T* for MBA40. The temperature is as follows: 1000/*T* (K^−1^) = 3.861, 3.745, 3.643, 3.545, 3.452, 3.364, 3.282, 3.204, 3.130, 3.060, 2.994, 2.930, 2.870, 2.813.

Taking one of the better performing IGs, MBA40IL, a further study scrutinizes the dielectric properties to better understand the presence of two *E*_a_. The dissipation factor tan δ (= ε”/ε’) is interesting to evaluate since it represents the ratio between the electrical power dissipated in the sample and the total power in the circuit [53]. With increasing temperature a large hump is observed due an increase in the dielectric properties. This signal stems from interfacial phenomena within the bulk of MBA40IL. The peak maximum in intensity (ω·τ = 1) is the relaxation time (Figure 14a) [54], and their positions are plotted against the inverse of the temperature (Figure 14b). Once again, an Arrhenius-type behavior with the presence of two defined slopes is observed. The associated energy of dielectric relaxation can be obtained as:


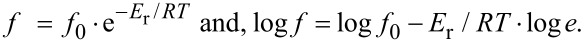


*E*_r_ = 0.72 eV in the lower temperature range and *E*_r_ = 0.38 eV in the higher temperature window. It is likely that the same charge carriers are responsible for the conductivity and dielectric properties. The slight deviation between the energy of activation and the energy of dielectric relaxation is probably due to the inner structure with the migration in the vicinity of silicate walls compared to the porous structure, as already observed in other composites [55]. The relative ion conductivity considering the physical versus chemical contributions was recently addressed in polymer-ionic liquid pairing [56].

**Figure 15:**
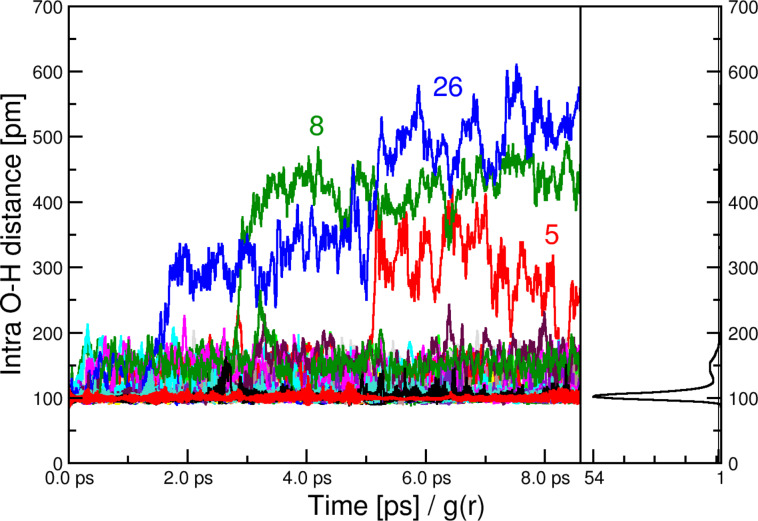
Full temporal development (from equilibrium search to production run) of all 32 intramolecular O–H distances in the SO_3_H group (left) along with the radial pair distribution function of the same atom pair (right). The numbers refer to the molecules in the simulation box.

To further evaluate the molecular processes occurring in the IGs, we have performed computational studies on the pure IL [BmimSO_3_H][PTS]. Figure 15 shows that there are three states of the SO_3_H-proton in its intramolecular O–H bond (the bond is described in [25]). The first state is a tight O–H bond of approximately 100 pm in length. Next there is a more loosely bound (150 pm) state and thirdly we find three protons that fully depart their parent SO_3_ group of the cation.

**Figure 16:**
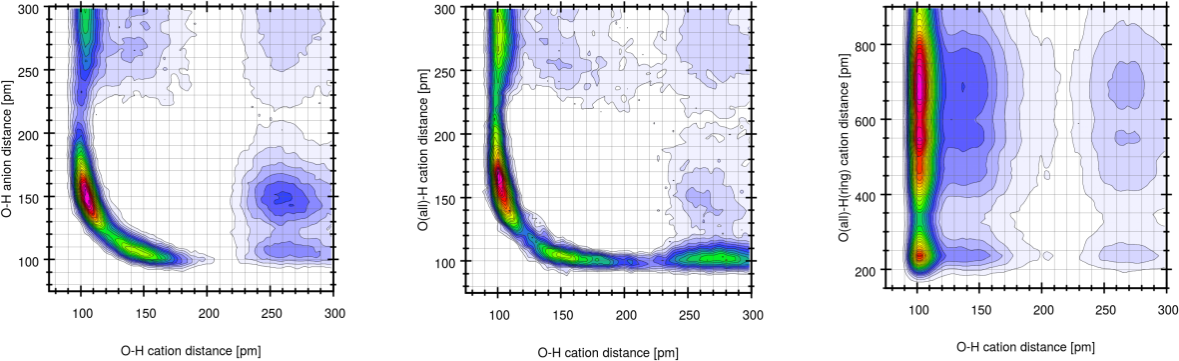
Combined distribution function of the intramolecular O–H distances (including only the bonding oxygen) on the *x*-axis. The *y*-axis is (from left to right) the O(anion)–H distance, the O(cation)–H distance (including all oxygen atoms of the SO_3_H group), and the O(cation)–H(ring) distances, respectively.

To elucidate whether these states are correlated to oxygen atoms of other cations or anions Figure 16 shows three combined distribution functions. These data clearly show that the proton – while normally bound to one oxygen atom – can form very short (150–180 pm) hydrogen bonds to the SO_3_ group of either other anions or other cations, see red areas in CDFs of Figure 16. If the hydrogen increases the intramolecular bond to approximately 150 pm either a cation SO_3_ group or one of the tosylate anions is close. A very large O–H distance of a former intramolecular O–H group can correlate with both, a short O–H anion or cation distance.

Moreover, inspection of the trajectory clearly shows that the protons are very mobile and several events can happen. For example, the proton of the SO_3_H can move to the SO_3_H group of another cation or to the SO_3_ group of an anion. This can be supported by a concerted proton transfer from another SO_3_H group or furthermore it can be supported by a sizeable hydrogen bond of the imidazolium ring proton towards the SO_3_H group. This is also reflected in Figure 16 (right) where short (<200 pm) H–O(SO_3_H) distances are observed while the original O–H intramolecular bond is still intact.

## Discussion

IGs are emerging materials with a high application potential [5,12–13]. Among others, silica has attracted interest as an IG matrix. This is due to the fact that silica is highly tunable in terms of the pore sizes, pore volumes, pore organization, and pore wall functionality [45–46]. These parameters enable the tuning of the IL loading, the IL/matrix ratio, and in some cases also the tuning of the IL behavior by tailoring the interactions of the IL with the wall of the silica [36,57]. Indeed, several research groups have provided different approaches towards silica-based IGs [11,16–18,22,24–25]. The main issue with most of these materials is that they cannot withstand mechanical stress and there is thus a need for stabilizing silica IGs for, e.g., flexible devices.

The current article describes new organosilica matrix materials based on silica and an organic linker. The linker provides the matrix with a rubberlike appearance rather than a high stiffness. Although the mechanical properties are still under investigation, the current data show that the organosilica matrix materials exhibit much higher stabilities towards drying and shrinking than conventional silica monoliths. While the latter often break upon drying, the current monoliths do shrink somewhat but remain intact over the whole composition range (Figure 2). This clearly shows that organosilica matrix materials are attractive host materials for the generation of advanced silica-based IGs.

Moreover, analysis of the organosilica materials with EA (Figure 3), TGA (Figure 4), IR spectroscopy (Figure 5), nitrogen sorption (Figure 6 and Table 1), and SAXS (Figure 6 and Table 1) shows that the properties of the organosilica monoliths can be tuned by a careful selection of the precursors, the precursor ratio, and the solvent used for preparation. Monoliths prepared in methanol are colorless and translucent and are therefore potentially interesting for optical devices or spectroscopy. On the other hand, methanol-based monoliths appear slightly softer when touching them and much more turbid in appearance. These materials may therefore be of interest as IL hosts for applications where a more flexible material without specific optical properties is desired, such as batteries or fuel cells.

The IL used in the current study (Scheme 1) is a new IL based on a related example published earlier [25]. The difference between the two ILs is the chain length of the sulfonated side chain attached to the imidazolium ring: while Delahaye et al. used a sulfopropyl chain, the current IL is functionalized with a sulfobutyl chain. The reason is the rather high toxicity of the 1,3-propanesultone precursor used for the synthesis of the previous IL. 1,3-Butanesultone, which was used in the current study has a similar reactivity but is less toxic. Moreover, the current IL, [BmimSO_3_H][PTS], has a significant advantage in terms of its thermal behavior because it does not crystallize. In contrast to the propane sultone analogue, which slowly crystallizes after 12 months [25], the longer alkyl chain of [BmimSO_3_H][PTS] suppresses crystallization of the IL even after over three years at ambient conditions and DSC data reproducibly show a glass transition at around −14 to −9 °C (Figure 11).

These data indicate that replacing the propyl with a butyl spacer provides two advantages, (i) a less hazardous synthesis protocol and (ii) efficient stabilization of the (highly viscous) liquid phase over extended periods of time. As a result, [BmimSO_3_H][PTS] is a viable candidate for use in membranes, especially when considering the simple, near quantitative synthesis (Figure 7) and the high thermal stability of the IL (Figure 10).

The combination of the organosilica matrix with the IL yields IGs that combine the appearance of the matrix with the conductivity of the IL. All IGs are stable and do not show degradation at ambient condition for over 6 months (Figure 7). IR spectroscopy (Figure 8) shows that the IGs are homogeneous in the sense that all IR spectra of the IGs are a superposition of the spectra of the matrix and the IL. The fact that the IR spectrum of the IL dominates the IG spectrum shows that the IG is mainly composed of the IL and that the matrix is the minority phase. This is confirmed by EA (Figure 9) and TGA (Figure 10). EA detects up to ca. 48% of carbon and TGA finds a residual mass of ca. 10% at 800 °C, indicating that ca. 90% of the material consist of IL and organic linker of the organosilica matrix. High IL loadings in silica IGs are quite common, although ca. 90% of IL is on the higher end of what has been reported so far [23–24]. These data therefore show that all organosilica monoliths yield stable monolithic IGs with high IL loading.

DSC data (Figure 11) and EIS analysis (Figure 12-14) show that (i) the confinement of the IL in the matrix does not affect its physical properties and (ii) that the conductivities in the IGs are comparable to those observed in other IGs [19,23–25,36]. In all cases DSC only finds a glass transition, which is independent both of the matrix type (TBA, TBM, MBA, MBM) and also of the fraction of IL present in the IGs.

EIS analysis (Figure 12-14) always finds an Arrhenius-type temperature dependence associated to high ion conductivity. Indeed an optimized composition, MBA40IL, exhibits an ionic conductivity of almost 10^−2^ S·cm^−1^ at 357 K (84 °C, the upper limit in this study). Figure 12 however shows that 84 °C is not the upper limit for this system. This is relevant for application in energy storage devices. Indeed, there is a correlation between the DSC and the EIS data in the sense that DSC finds a *T*_g_ at around −9 °C and EIS finds a change in the Arrhenius behavior at around 10 °C. Although there is a slight difference between these two measurements, the data indicate a change in mobility in the general temperature window between −10 and +10 °C. The difference in the transition temperatures is likely due to the quite different experimental setups, but this will have to be verified with a modified EIS setup in the future.

Possibly, the rather high conductivity of the IGs is also due to the fact that the organic bridging moiety in the organosilica matrix contains a nitrogen atom that may also be protonated because alkylamines have p*K*_a_ values between 9 and 10. As a result, we speculate that protons can not only transfer between individual sulfonate groups, but also to amino groups of the linker and on to other sulfonates from there. This effect may contribute to the rather efficient conduction but the details are currently under investigation using solid-state NMR spectroscopy.

The hypothesis of very mobile protons is however qualitatively supported by computer simulations (Figure 15 and Figure 16). The simulation data clearly show that significant proton transfer can occur between many different atoms of both the cations and the anions of the IL. This suggests that other proton-accepting atoms such as the nitrogen atoms in the bridge elements of the silica matrix will also be efficient proton acceptors. In turn, a contribution of these positions will likely also contribute to the overall conductivity of the materials.

These data indicate that in the current IGs, the interaction of the IL with the matrix is weaker than in some previously reported IGs [36,57]. This is surprising, because previous data indicate that an organic modification of a silica host can very strongly affect the behavior of a confined IL in a silica matrix [57]. The reason for this weak interaction in the current materials is not clear, but we speculate that the zwitterionic nature of the [BmimSO_3_H][PTS] IL will lead to very strong interactions within the IL rather than between the IL and the silica pore wall. The main advantage of this behavior is that the IL is “self-sufficient” as far as the ionic transport is concerned and transport properties will likely not change significantly over extended periods of time because the IL does not crystallize or interact strongly with the surrounding host material.

Overall, the current study shows that the combination of [BmimSO_3_H][PTS] with a suitable organosilica host provides access to robust and somewhat flexible IGs that may be interesting as membrane materials in electrochemical devices such as sensors, batteries, or fuel cells.

## Conclusion

The current article is the first account on organosilica host materials for ionogel fabrication. Compared to pure silica host materials, organosilica hosts offer many advantages such as better mechanical stability, the possibility to tune the composition (and hence possibly the properties), and the possibility to include organic moieties in the host material that may enhance some properties such as proton conduction. Overall, organosilica IGs are therefore interesting materials with a high application potential in various fields.
